# Knockdown of the Expression of Two Trehalase Genes with RNAi Disrupts the Trehalose and Chitin Metabolism Pathways in the Oriental Armyworm, *Mythimna separata*

**DOI:** 10.3390/insects15030142

**Published:** 2024-02-21

**Authors:** Hongjia Yang, Yixiao Wang, Weijia Zhang, Xinxin Zhang, Sibo Wang, Mengyao Cui, Xiaohui Zhao, Dong Fan, Changchun Dai

**Affiliations:** Department of Plant Protection, College of Plant Protection, Northeast Agricultural University, Harbin 150036, China; 18800426621@163.com (H.Y.); 15663583528@163.com (Y.W.); 15504575201@163.com (W.Z.); xinxinz@neau.edu.cn (X.Z.); 13796228303@163.com (S.W.); 15303645543@163.com (M.C.); zhaoxiaohui3521@163.com (X.Z.)

**Keywords:** *Mythimna separata*, trehalase, trehalose, chitin, RNAi

## Abstract

**Simple Summary:**

Trehalose is the most important carbohydrate in insects. It is required for chitin synthesis and, thus, insect growth and development. Trehalase is the only enzyme that catalyzes the decomposition of trehalose. *Mythimna separata* is an important pest of cereal crops. We cloned and identified *Tre1* and *Tre2* cDNA sequences in *M. separata*. Analysis of *MsTre1* and *MsTre2* expression revealed that *MsTre1* was highly expressed in the midgut, and *MsTre2* was highly expressed in the integument. The expression of *MsTre1* and *MsTre2* was the highest in the pupal stage. We used RNA interference to inhibit *MsTre1* and *MsTre2* expression. *MsTre1* and *MsTre2* silencing resulted in significant changes in the expression of genes associated with trehalose and chitin metabolism, and significantly reduced the MsTre1 and MsTre2 activity and the glucose and chitin content. Hematoxylin and eosin staining, and transmission electron microscopy showed that the silencing of *MsTre1* slowed larval molting, and the new cuticle was significantly thinner in ds*MsTre1*-injected larvae than in control larvae. Overall, *MsTre1* and *MsTre2* are two effective genes in *M. separata* that regulate insect growth via the trehalose and chitin metabolism pathways, and *MsTre1* is more important for cuticle formation in the epidermis than *MsTre2*.

**Abstract:**

Trehalose is an important carbohydrate substance in insect hemolymph. Chitin is the main component of cuticle and peritrophic matrix in insects. Trehalase (Tre) catalyzes the decomposition of trehalose. Few studies of trehalase in lepidopteran insects have been conducted. Here, the functions of soluble Tre (Tre1) and membrane-bound Tre (Tre2) in the growth and development of *Mythimna separata* were investigated. We cloned and identified *Tre1* and *Tre2* cDNA sequences in *M. separata*. Analysis expression revealed that *MsTre1* and *MsTre2* were highly expressed in midgut and integument, respectively. The expression of *MsTre1* and *MsTre2* was highest in the pupal stage. We used RNA interference (RNAi) to inhibit *Tre* expression in *M. separata* larvae. Injection of ds*MsTre1* or ds*MsTre2* resulted in abnormal phenotypes and impeded normal molting. Silencing of *MsTre1* and *MsTre2* resulted in significant changes in the expression of genes in the trehalose and chitin metabolism pathways, significantly increased the trehalose and glycogen content, and significantly decreased MsTre1 and MsTre2 activity, the glucose content, and the chitin content in midgut and integument. Silencing of *MsTre1* slowed larval molting, and the new cuticle was significantly thinner. These results indicate that RNAi of *Tre* may be useful for control strategies against *M. separata*.

## 1. Introduction

Trehalose is a disaccharide composed of two glucose molecules and is widely distributed in insects, fungi, bacteria, yeast, invertebrates, and plants. In insects, trehalose is essential for various biological processes, including energy metabolism, recovery from stress, and chitin synthesis [1,2,3]. Trehalose accounts for about 90% of the total sugar in insect hemolymph, and can be used as an energy substance to supply energy for various biological processes of insects [4,5,6,7,8]. It can be synthesized in large quantities under adverse conditions to provide protection against environmental stress; it also plays a key role in the molting and metamorphosis of insects [8,9]. Trehalose metabolism in insect hemolymph is essential for many physiological processes of insects, including flight, diapause, and molting [10,11].

Trehalase (Tre) is the only glycosidase that can specifically break down trehalose into two molecules of glucose [12,13]. Two distinct forms of Tre exist in insects, soluble Tre (Tre1) and membrane-bound Tre (Tre2). The first insect *Tre1* gene was cloned from *Tenebrio molitor* in 1992 [14]. However, it was not until 2005 that the first insect *Tre2* gene from *Bombyx mori* was cloned [15]. The main difference between Tre1 and Tre2 is that Tre2 generally has a transmembrane domain. The spatio-temporal expression patterns of *Tre1* and *Tre2* differ in various insects [16,17]. Tre1 is an intracellular enzyme that mainly occurs in the digestive system and circulatory system of insects; it is responsible for breaking down the trehalose in insect cells. Tre2 is an extracellular enzyme that mainly occurs in the basal membrane or microvilli; it is mainly responsible for the decomposition of exogenous trehalose [7,18,19]. Trehalase genes have been identified in a variety of insects, such as *Drosophila melanogaster* [13], *Aphis glycines* [18], *Apolygus lucorum* [19], *Omphisa fuscidentalis* [20], *Laodelphax striatellus* [21], *Nilaparvata lugens* [22]. The functions of the Tre1 and Tre2 in insects are also different. In *Spodoptera exigua*, *SeTre1* was mainly responsible for chitin synthesis in the cuticle, and *SeTre2* was mainly responsible for chitin synthesis in the midgut [23]. In *Bemisia tabaci*, *BtTre2* played a more critical role during development, while *BtTre1* may be involved in damage to plant defense [24].

Chitin is composed of N-acetylglucosamine, which is the main component comprising the peritrophic matrix and cuticle of insects; it plays an important role in maintaining the structure and the permeability barrier of insects [25]. Chitin is regularly synthesized and metabolized in insects to ensure normal molting and support normal growth and development [26,27,28]. The biosynthesis of chitin begins with trehalose [29], which is a highly complex physiological and biochemical process involving eight enzymes: Tre, hexokinase (HK), glucose-6-phosphate isomerase (G6PI), glutamine-fructose-6-phosphate aminotransferase (GFAT), glucosamine-6-phosphate N-acetyltransferase (GNAT), phosphoacetylglucosamine mutase (PGM), UDP-N-acetylglucosamine pyrophosphorylase (UAP), and chitin synthase (CHS) [20,21,30,31,32]. Chitinase (Cht) is a key enzyme in the chitinolytic pathway [22]. Tre, which is the first and key enzyme in the chitin synthesis pathway, is essential for chitin synthesis in insects. Previous studies of Tre have mainly centered around the importance of trehalose in the growth and development of insect and the use of molecular biological methods to interfere with the expression of *Tre* genes. For example, the *Tre* of *Diaphorina citri* was silenced by RNA interference (RNAi), and this affected chitin metabolism and thus growth and development [33]. Trehalose metabolism has been shown to regulate chitin metabolism in some hemipterans [33,34]. Trehalose, as a precursor of chitin biosynthesis, which can directly affect the synthesis and hydrolysis of chitin, and thus affect the molting in insects [22]. Knockdown of *Ldtre1* and *Ldtre2* resulted in weight loss, increased trehalose content, and impaired chitin synthesis in *Leptinotarsa decemlineata* [35]. Therefore, genes related to trehalose and chitin metabolism pathways are considered promising molecular targets for pest control. However, few studies of trehalose metabolism in lepidopteran insects have been conducted, and the expression and functions of the related genes in trehalose metabolism pathway in lepidopteran insects require further study.

The oriental armyworm, *Mythimna separata* (Walker) (Lepidoptera: Noctuidae), is an important agricultural pest with strong migratory behaviors and omnivorous feeding habits [36,37,38,39]. *M. separata* mainly feeds on cereal crops such as corn, wheat, and rice, and leads to substantial reductions in yield. These crops comprise a major portion of the food supply; there is thus an urgent need to control populations of these pests [40]. The prolonged use of chemical insecticides has facilitated the evolution of resistance to several pesticides in insects; improved pest management methods are needed to prevent the evolution of resistance and other environmental problems [41,42,43]. We previously characterized the role of MsTPS in *M. separata* trehalose biosynthesis and its effect on chitin synthesis and growth and development [44]. However, Tre (MsTre) has not been functionally characterized in *M. separata*. Here, the role of MsTre1 and MsTre2 in the decomposition of trehalose, chitin metabolism, and molting of *M. separata* were clarified through gene cloning, sequence analysis, analysis of spatial-temporal expression patterns, and RNAi. Our results reveal that the *Tre* genes required for trehalose breakdown provide effective targets for the control of *M. separata* by RNAi. These results deepen our understanding of the role of trehalose in *M. separata* and will aid the development of improved control methods.

## 2. Materials and Methods

### 2.1. Insects

*M. separata* was initially derived from Xiangyang Station (Harbin, China), and reared at 25 °C, 70% humidity, and 14 h light:10 h dark photoperiod for several generations. Larvae and adults were fed with fresh corn seedlings and 5% honey water, respectively.

### 2.2. Identification of MsTre1 and MsTre2

Assemblies of the *MsTre1* and *MsTre2* transcript sequences were identified by searching the *M. separata* transcriptome database (NCBI Accession ID: PRJNA919163) (Annoroad, Beijing, China). The total RNA was extracted from 4th-instar larvae using TRIzol (Invitrogen, Carlsbad, CA, USA), and 1st strand cDNA was synthesized using the PrimeScript^TM^ 1st Strand cDNA Synthesis Kit (TaKaRa, Beijing, China). According to the screened sequences, the primers *MsTre1*-F, *MsTre1*-R, *MsTre2*-F, and *MsTre2*-R were designed using the Primer Premier 5.0 software for PCR amplification (Table S1). The products were purified using the Gel Extraction kit (Omega, Norcross, GA, USA) and inserted into the pMD^TM^18-T Vector (TaKaRa, Beijing, China), and sequenced to confirm their accuracy.

### 2.3. Bioinformatic Analysis and Phylogenetic Tree Construction of MsTre1 and MsTre2

The *MsTre1* and *MsTre2* sequences were registered in the NCBI database. The DNAMAN 9.0 software was used to conduct sequences alignment of *MsTre1* and *MsTre2*. The conserved domain was detected using the SMART program (http://smart.embl-heidelberg.de/ (accessed on 30 April 2022)). The molecular weight and isoelectric point were predicted using the Expasy Compute pI/Mw (https://www.expasy.org/ (accessed on 30 April 2022)). A phylogenetic tree from Tre of different insects was constructed with MEGA 7.0 software using the maximum likelihood method [45].

### 2.4. Spatial-Temporal Expression Patterns of MsTre1 and MsTre2

RNA was extracted from samples collected during different developmental stages, including first-day of 50 eggs, 10 first-to-second-instar larvae, 2 third-to sixth-instar larvae, 2 pupae, and 2 adults, and from different tissues of 20 larvae including foregut, midgut, hindgut, fat body, salivary gland, Malpighian tubules, and integument. Reverse transcription was performed using the same methods as described above. The spatial-temporal expression patterns of *MsTre1* and *MsTre2* were determined using quantitative Real-Time PCR (RT-qPCR).

The RT-qPCR reaction system contained the following components: 2 µL of cDNA, 0.8 µL each of sense and anti-sense primers, 10 µL of SYBR RT-qPCR Mix (Toyobo, Shanghai, China), and 6.4 µL of ddH_2_O. The mixed sample plate was placed into PCR instrument (Thermo Scientific, Waltham, MA, USA). The melting curves were assessed to test the purity of the RT-qPCR reaction. *Beta-actin* (*Msβ-actin*) and *glyceraldehyde-3-phosphate dehydrogenase* (*MsGAPDH*) were used as reference genes. The RT-qPCR primers used are listed in Table S1. The data were analyzed using the 2^−ΔΔCT^ method [46]. Each treatment contained three technical replicates and three biological replicates.

### 2.5. Double-Stranded RNA (dsRNA) Preparation and RNAi of MsTre1 and MsTre2

dsRNAs of the *MsTre1* and *MsTre2* were prepared using a MEGAscript^®^ RNAi Kit (Thermo Scientific, Waltham, MA, USA). Specific primers targeting *MsTre1* and *MsTre2* were designed using E-RNAi (http://www.dkfz.de/signaling/e-rnai3/idseq (accessed on 1 August 2022)) [47]. The effective siRNAs sites of ds*MsTre1* and ds*MsTre2* were predicted by the DNAMAN 9.0 software and the siRNA Wizard tool (https://www.invivogen.com/sirnawizard/design.php (accessed on 29 August 2022)) [48]. The dsRNA concentration was measured using a spectrophotometer (Thermo Scientific, Massachusetts, America), and dsRNA quality was confirmed by 1% agarose gel electrophoresis. The first-day fourth-instar larvae were injected with 2 µL of 2 µg µL^−1^ dsRNA for *MsTre1* or *MsTre2* using a microsyringe. ds*GFP* was used as a control. The larvae were normally fed fresh corn leaves after injection, and were collected at 6, 12, 24, 48, and 72 h after injection for subsequent analysis. In addition, larvae were collected at 6, 12, 24, and 48 h after RNAi and dissected in normal saline to obtain midgut and integument to detect chitin content. The expression levels of target genes and other related genes from two larvae after RNAi were determined using RT-qPCR to assess the effect of RNAi. Each treatment contained three technical replicates and three biological replicates. The ds*MsTre1*, ds*MsTre2*, and the effective siRNAs sites were marked in Figure S1. The dsRNA and RT-qPCR primers used are listed in Table S1.

### 2.6. Determination of the MsTre1 and MsTre2 Activity, Sugar, and Chitin Content

To determine the MsTre1 and MsTre2 activity, sugar, and chitin content, the larvae were collected at 6, 12, 24, and 48 h after injection of dsRNA. The MsTre1 and MsTre2 activity was determined as described previously, with some modifications [20,22,49]. Five larvae were homogenized in PBS (pH 7.2) (Sangon, Shanghai, China), and sonicated for 30 s (Sxsonic, Shanghai, China). The homogenates were then centrifuged at 30,000× *g* at 4 °C for 1 h (Beckman, Brea, CA, USA). The resulting supernatant was used to determinate the MsTre1 activity and the protein content; the precipitate was suspended in PBS for measurements of MsTre2 activity and the protein content. The protein concentrations were determined as previously described using the protein-dye binding method [20,50]. Next, 225 µL of the above supernatant or suspension and 75 µL of 40 mM trehalose were added to a centrifuge tube and incubated for 1 h at 37 °C. They were then centrifuged at 12,000× *g* for 10 min at 4 °C, and the Tre activity was detected with 10 µL supernatant using a Glucose Assay kit (Sangon, Shanghai, China) [51].

The trehalose content was estimated according to a previously described method [52,53], with slight modifications [44], three larvae per group. The glucose and glycogen content were determined using Glucose and Glycogen Assay kit (Sangon, Shanghai, China), respectively, three larvae per group. The chitin content in midgut and integument of twenty larvae was determined by a previously reported method [35,44,54,55]. Each measurement was conducted using three biological replicates.

### 2.7. Microsectioning and Hematoxylin and Eosin (H&E) Staining of the Cuticle

To further explore the effects of injecting ds*MsTre1* and ds*MsTre2* on cuticle, we performed H&E staining [56]. We dissected the cuticle of the larvae at 12, 24, 48, and 72 h after injection of ds*MsTre1*, ds*MsTre2*, or ds*GFP*. The samples were fixed with 4% paraformaldehyde at 4 °C for 48 h, then dehydrated with ethanol and xylene, and embedded with paraffin at −20 °C. The paraffin block was cut to 4 µm with a microtome (Leica, Shanghai, China) and then stained with H&E. The stained sections were visualized and photographed using Pannoramic scanner (3D Histech, Budapest, Hungary).

### 2.8. Transmission Electron Microscopy (TEM) of the Cuticle

TEM was performed to analyze the ultrastructure of the cuticle after *MsTre1* and *MsTre2* knockdown [47,56]. The larvae were dissected and their cuticles were obtained at 72 h after injection of ds*MsTre1*, ds*MsTre2*, or ds*GFP*. Five larvae were collected from each group, and the cuticles were cut into small pieces no larger than 1 cubic millimeter, and they were fixed using 2.5% glutaraldehyde for 2 weeks at 4 °C. The samples were then dehydrated with ethanol, impregnated with acetone, and embedded with resin. The section was cut to 50 nm, and was observed and captured using a H-7650 transmission electron microscope (Hitachi Ltd., Tokyo, Japan).

### 2.9. Effects on Growth and Development after MsTre1 and MsTre2 Knockdown

The fourth-instar larvae were injected with 2 µL of ds*MsTre1*, ds*MsTre2*, or ds*GFP*. The body length and weight, feeding amount, molting rate, and mortality in each group were continuously monitored for 3 days at a 24 h interval. In total, 3 replicates of each treatment were performed, with 30 larvae per group. The insects showing abnormal development were photographed and analyzed using Helicon Focus 8.1.0 and Helicon Remote 4.4.4 software.

### 2.10. Statistical Analysis

GraphPad Prism 9.8.0 software was used for statistical analysis and plot results. One-way ANOVA was used to identify the significance of differences among groups using Tukey’s test (*p* < 0.05). Resulting pairs were compared using Student’s *t*-test. All data are shown as means ± SE from at least three biological replicates.

## 3. Results

### 3.1. Bioinformatic Analysis of MsTre1 and MsTre2

The cDNA sequences of *MsTre1* (MN894706) and *MsTre2* (MN894707) were obtained from the *M. separata* transcriptome database. The result of the sequence alignments shows that the identity between *MsTre1* and *MsTre2* was 47.87% (Figure S1). The open reading frame of *MsTre1* comprised 1755 nucleotides encoding 584 amino acids, and the open reading frame of *MsTre2* comprised 1938 nucleotides encoding 646 amino acids. The isoelectric points of MsTre1 and MsTre2 were 4.64 and 6.05, respectively, and the molecular weights were 66.02 and 73.89 kDa, respectively. They all contained the Tre-conserved domains.

Phylogenetic analysis revealed that Tre1 and Tre2 were in two different clusters, which indicated that Tre1 and Tre2 are two different proteins. MsTre1 clustered first with Tre1 of *Helicoverpa armigera*, *Operophtera brumata*, and *S. exigua* and last with Tre1 of *Papilio machaon*, *Papilio xuthus*, and *Plutella xylostella*. MsTre2 clustered first with Tre2 of *Helicoverpa zea*, *Mythimna loreyi,* and *S. exigua*, and last with Tre2 of *Rondotia menciana*, *B. mori*, and *Leptidea sinapis* (Figure S2).

### 3.2. Spatio-Temporal Expression Patterns of MsTre1 and MsTre2

**Figure 1:**
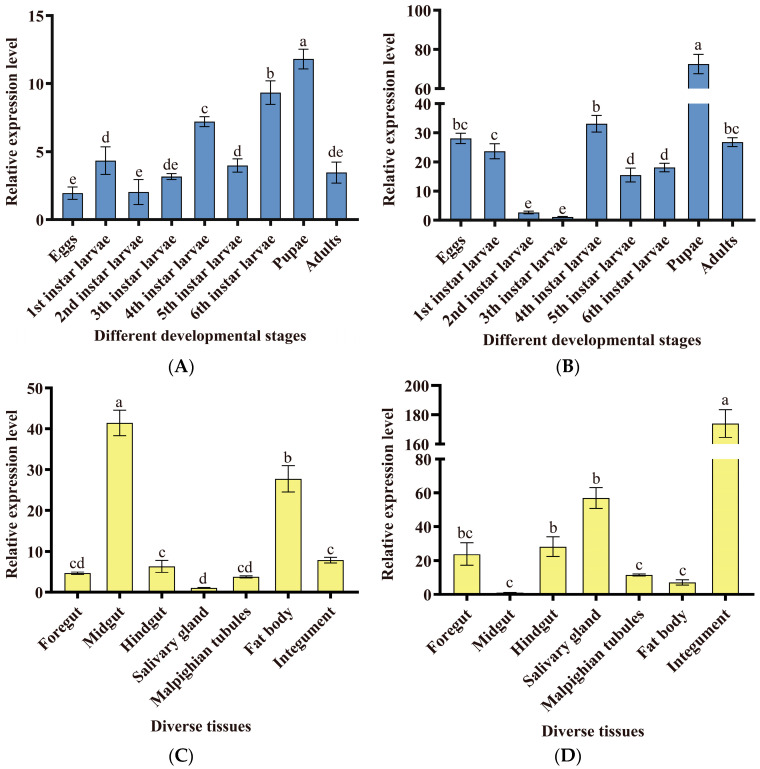
Spatio-temporal expression patterns of *MsTre1* and *MsTre2* at different developmental stages ((**A**) *MsTre1*, (**B**) *MsTre2*)) and in diverse tissues ((**C**) *MsTre1*, (**D**) *MsTre2*)). Different letters indicate significant differences (*p* < 0.05) according to Tukey’s multiple comparison test.

We analyzed the spatio-temporal expression patterns of *MsTre1* and *MsTre2* by RT-qPCR. The results showed that *MsTre1* and *MsTre2* were expressed at all developmental stages. However, the expression patterns of *MsTre1* and *MsTre2* were different. The expression of *MsTre1* and *MsTre2* was highest in the pupal stage. The expression of *MsTre1* was lowest in eggs, and the expression of *MsTre2* was lowest in third-instar larvae (Figure 1A,B). *MsTre1* and *MsTre2* were expressed in all the examined tissues. The expression of *MsTre1* was highest in the midgut, followed by the fat body; its expression was lowest in the salivary gland. The expression of *MsTre2* was highest in the integument and lowest in the midgut (Figure 1C,D).

### 3.3. The Expression of MsTre1, MsTre2, and MsTPS, Trehalase Activity and Sugar Content after RNAi

**Figure 2:**
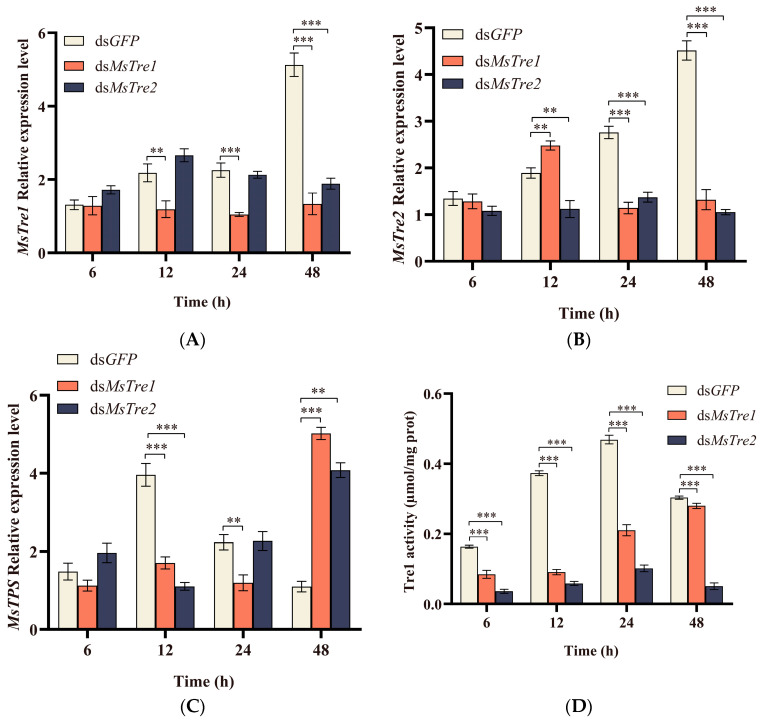

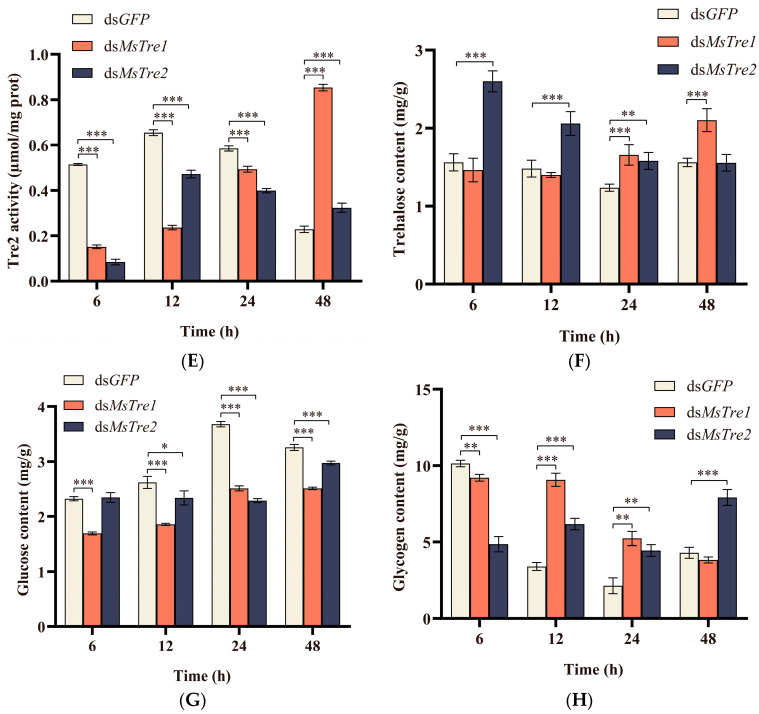
Effects of *MsTre1* and *MsTre2* RNAi treatment on the *MsTre1* (**A**), *MsTre2* (**B**)*,* and *MsTPS* (**C**) expression, MsTre1 (**D**) and MsTre2 (**E**) activity, and the trehalose (**F**), glucose (**G**), and glycogen (**H**) content at different time points. Statistical analyses were performed using *t*-tests, and asterisks indicate significant differences compared with the respective controls (* *p* < 0.05, ** *p* < 0.01, and *** *p* < 0.001).

We characterized the expression of *MsTre1*, *MsTre2*, *MsTPS*, MsTre1, and MsTre2 activity, and concentrations of trehalose, glucose, and glycogen at 6, 12, 24, and 48 h after injection of ds*MsTre1* and ds*MsTre2*. The results showed that the expression of *MsTre1* was significantly inhibited when ds*MsTre1* was injected at 12, 24, and 48 h; *MsTre1* was most efficiently silenced at 48 h and the inhibition rate was 73.91%. Besides, the *MsTre2* expression was significantly decreased at 48 h after injection of ds*MsTre1* (Figure 2A). In addition, *MsTre2* expression was significantly inhibited when ds*MsTre2* was injected at 12, 24, and 48 h; *MsTre2* was most efficiently silenced at 48 h and the inhibition rate was 76.67%; and the *MsTre1* expression was significantly increased at 12 h and significantly decreased at 24 and 48 h after injection of ds*MsTre2* (Figure 2B). Injection of dsRNA significantly inhibited the expression of *MsTre1* and *MsTre2*, indicating that follow-up studies could be conducted.

The *MsTPS* expression decreased significantly at 12 h after ds*MsTre2* injection and increased significantly at 48 h (Figure 2C). The *MsTPS* expression decreased significantly at 12 and 24 h after ds*MsTre1* injection and increased significantly at 48 h. Figure 2D shows that injection of both ds*MsTre1* and ds*MsTre2* resulted in a significant decrease in MsTre1 activity at 6, 12, 24, and 48 h. Furthermore, injection of ds*MsTre1* and ds*MsTre2* led to a significant decrease in MsTre2 activity at 6, 12, and 24 h and a significant increase at 48 h (Figure 2E). The trehalose content was increased significantly and the glucose content was decreased significantly after ds*MsTre1* and ds*MsTre2* injection. In addition, injection of ds*MsTre1* and ds*MsTre2* led to a significant decrease in glycogen content at 6 h and a significant increase at 12 h and 24 h (Figure 2F–H).

### 3.4. Alteration in the Chitin Content and Expression of Genes in the Chitin Metabolism Pathway after RNAi

**Figure 3:**
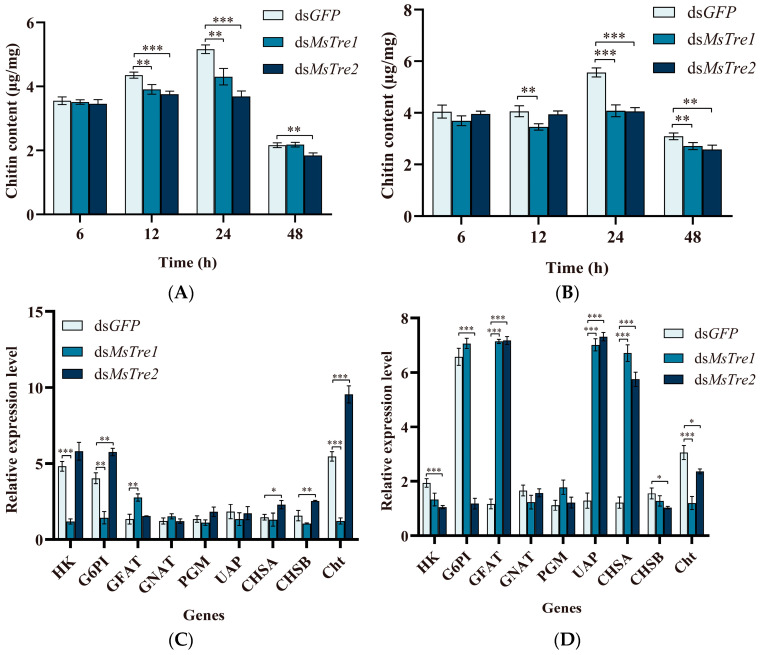

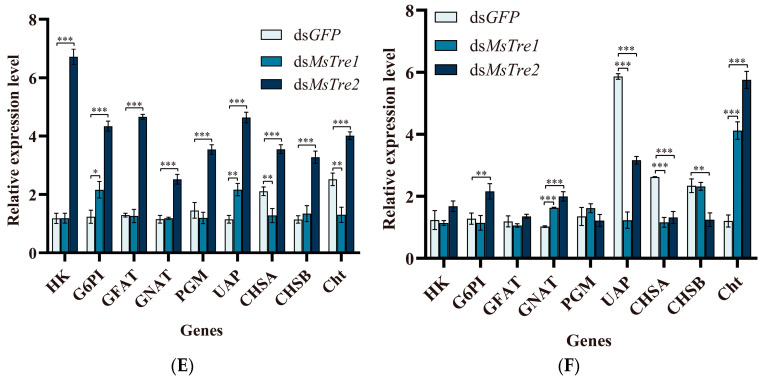
Effect of *MsTre1* and *MsTre2* knockdown on the chitin content and expression levels of related genes in *M. separata*. Chitin content in integument (**A**) and midgut (**B**) after injection of ds*MsTre1* and ds*MsTre2*. The expression levels of genes involved in chitin metabolism after injection of ds*MsTre1* and ds*MsTre2* at 6 (**C**), 12, (**D**) 24 (**E**), and 48 h (**F**). Statistical analyses were performed using *t*-tests, and asterisks indicate significant differences compared with the respective controls (* *p* < 0.05, ** *p* < 0.01, and *** *p* < 0.001).

To determine whether the chitin content of *M. separata* is affected by *MsTre1* and *MsTre2*, dsRNA-injected larvae were dissected to obtain the midgut and integument, and the chitin content of these tissues was determined. The chitin content of midgut significantly decreased at 12 and 24 h after injection of ds*MsTre1* and ds*MsTre2* (Figure 3A). The chitin content of integument significantly decreased at 24 and 48 h after injection of ds*MsTre1* and ds*MsTre2* (Figure 3B).

To investigate the effect of *MsTre1* and *MsTre2* on the transcription of genes in the chitin metabolism pathway, we analyzed the expression of these genes after RNAi treatment. The fourth-instar larvae were injected with ds*MsTre1* or ds*MsTre2*, and the transcript levels were examined at 6, 12, 24, and 48 h after injection. *CHS* is a key gene for chitin synthesis. The injection of ds*MsTre1* resulted in a significant decrease in the expression of *MsCHSA* at 24 and 48 h and a significant increase at 12 h. Additionally, the expression levels of both *MsCHSA* and *MsCHSB* were significantly regulated by ds*MsTre2* injection. After 12 h of ds*MsTre2* injection, the *MsCHSA* expression increased significantly, and the *MsCHSB* expression decreased significantly. The expression levels of *MsCHSA* and *MsCHSB* increased significantly at 24 h after ds*MsTre2* injection and decreased significantly at 48 h (Figure 3C–F).

The other genes expression in the chitin biosynthesis pathway, including *MsHK*, *MsG6PI*, *MsGFAT*, *MsGNAT*, *MsPGM*, and *MsUAP*, was also analyzed by RT-qPCR. The expression levels of these genes were significantly altered several times at different time points after injection of ds*MsTre1* or ds*MsTre2*. Changes in *MsUAP* were significant; after injection of ds*MsTre1* and ds*MsTre2*, the *MsUAP* expression first decreased, increased, and then decreased (Figure 3C–F).

Cht can hydrolyze chitin. The expression of *MsCht* significantly decreased at 6, 12, and 24 h, and significantly increased at 48 h after *MsTre1* silencing (Figure 3C–F). The *MsCht* expression increased significantly at 6, 24, and 48 h, and decreased significantly at 12 h after *MsTre2* silencing.

### 3.5. Effect on M. separata Growth and Development after RNAi

**Figure 4:**
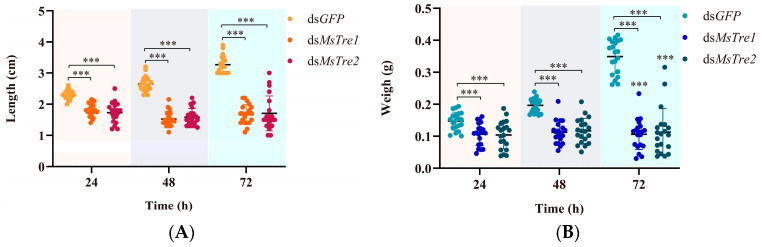

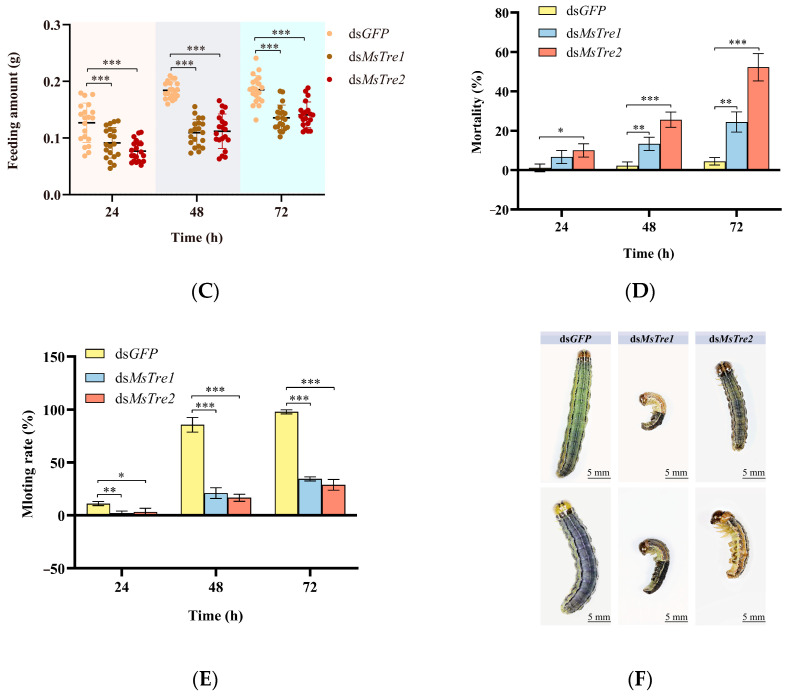
Effect of the silencing of *MsTre1* and *MsTre2* on the length (**A**), weight (**B**), feeding amount (**C**), mortality (**D**), and molting rate (**E**) of *M. separata* (**F**). Injection of ds*MsTre1* and ds*MsTre2* resulted in abnormal phenotypes of *M. separata*. Scale bars: 5 mm. Statistical analyses were performed using *t*-tests, and asterisks indicate significant differences compared with the respective controls (* *p* < 0.05, ** *p* < 0.01, and *** *p* < 0.001).

To explore the biological functions of *MsTre1* and *MsTre2* in the *M. separata* growth and development and molting process, ds*MsTre1*, ds*MsTre2*, and ds*GFP* were injected into the fourth-instar larvae. The length, weight, and feeding amount were significantly lower for larvae injected with ds*MsTre1* and ds*MsTre2* than for larvae injected with ds*GFP* at 24, 48, and 72 h after injection (Figure 4A–C). In addition, the mortality rate was significantly higher and the molting rate was significantly lower in ds*MsTre1*- and ds*MsTre2*-injected larvae than in ds*GFP*-injected larvae (Figure 4D,E). The phenotypes of *M. separata* were abnormal after injection of ds*MsTre1* and ds*MsTre2* at 72 h (Figure 4F). These results indicate that the knockdown of both *MsTre1* and *MsTre2* significantly affected the growth and development of *M. separata.*

### 3.6. Effects on Cuticle Formation after MsTre1 and MsTre2 Knockdown

**Figure 5:**
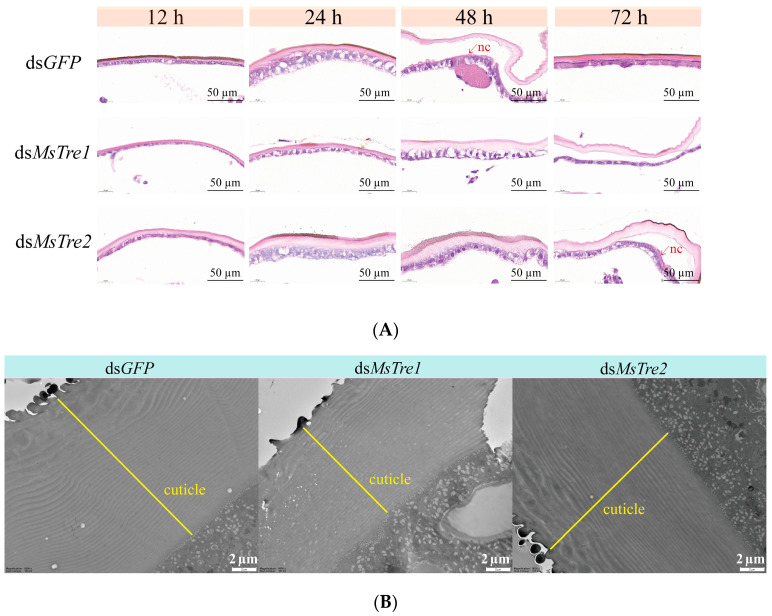
Cuticle formation after *MsTre1* and *MsTre2* knockdown. (**A**) Microsectioning and H&E staining of the integument after injection with ds*GFP*, ds*MsTre1*, and ds*MsTre2*. New cuticle (nc). Scale bars: 50 µm. (**B**) TEM analysis of the cuticle of ds*GFP*-, ds*MsTre1*-, and ds*MsTre2*-injected larvae. Scale bars: 2 µm.

We prepared stained sections with H&E of the integument to determine the effects of the knockdown of *MsTre1* and *MsTre2*. H&E staining results showed that molting was delayed after *MsTre1* or *MsTre2* knockdown. After molting, larvae injected with ds*GFP* and ds*MsTre2* had new cuticles, and larvae injected with ds*MsTre1* did not (Figure 5A). We performed TEM analysis to observe ultrastructural changes in the cuticle after *MsTre1* or *MsTre2* knockdown. The results showed that larvae injected with ds*MsTre1* had thinner cuticles and fewer cuticular layers than controls. There was no significant difference in the cuticles of ds*MsTre2*-injected larvae and control larvae (Figure 5B, Table S2).

## 4. Discussion

Trehalose metabolism is closely related to the energy supply, stress resistance, and chitin metabolism of insects [57,58]. In our study, we identified a soluble Tre (*MsTre1*) and membrane-bound Tre (*MsTre2*) based on a transcriptome search of *M. separata*. Our results demonstrate that MsTre1 and MsTre2 are essential for trehalose and chitin metabolism, and the growth and development of *M. separata*. These findings indicate that *MsTre1* and *MsTre2* serve as key regulators of trehalose and chitin metabolism in *M. separata*, and would provide effective target genes to control *M. separata*.

In our study, the expression level *MsTre1* and *MsTre2* was highest in the pupal stage (Figure 1A,B). In addition, *MsTre1* was highly expressed in the midgut and fat body; *MsTre2* was highly expressed in the integument (Figure 1C,D). In *Prodenia litura*, *PlTre1* and *PlTre2* were expressed in the fat body, midgut, trachea, and integument. The expression of *PlTre1* and *PlTre2* was higher in the third-instar larvae than in the other instars [59]. *SeTre2* was expressed in the fat body, Malpighian tubules and midgut in *S. exigua* [60]. *HaTre1* was highly expressed in the midgut of *H. armigera*, and its expression was lower in the trachea, Malpighian tubules, and head; *HaTre2* was highly expressed in the head and midgut [61]. Overall, the expression pattern of *MsTre1* and *MsTre2* was consistent with the *Tre1* and *Tre2* of other insects.

RNAi has been widely used for the screening of target genes for the control of pests [62,63]. In our study, the expression of *MsTre1* and *MsTre2* was successfully interfered, and the interference efficiency of *MsTre1* and *MsTre2* reached 73.91% and 76.67%, respectively, after 48 h (Figure 2A,B). *MsTre2* expression decreased significantly at 48 h after RNAi of *MsTre1* (Figure 2A). Previous studies have also found that knockdown of *SeTre1* or *SeTre2* expression leads to the up-regulation of the other *Tre* expression in *S. exigua* [64], suggesting that a compensatory regulatory mechanism might underlie the expression of *Tre1* and *Tre2* in insects. In this study, the expression levels of *MsTre1* and *MsTre2* decreased significantly 12 h after dsRNA injection, and the function of dsRNA began earlier. Similar results have been found in other lepidopterans, such as, In *Hyphantria cunea*, injection of ds*HcCht5* had a silencing effect on the target gene at 12 h [65]. Besides, the insects used were all fourth-instar larvae in RNAi, and in the larval stage of *M. separata*, the expression levels of *MsTre1* and *MsTre2* were highest in the fourth-instar larvae (Figure 1A,B). Studies also showed that the efficiency of RNAi was better when the expression levels of target genes were higher in *Spodoptera frugiperda* [66]. This may also be one of the reasons for the early function of dsRNA in this study, but there are many factors affecting the efficiency of RNAi which need to be further studied.

Silencing of *MsTre1* and *MsTre2* expression inhibited upstream *MsTPS* expression (Figure 2C). This might stem from the increased trehalose content associated with the disruption of trehalose decomposition; thus, *M. separata* might reduce the conversion of glucose to trehalose by reducing the expression of *MsTPS*. This finding is consistent with the studies of *Harmonia axyridis* and *Tribolium castaneum* showing that injection of *Tre1* and *Tre2* can inhibit the expression of *TPS* [23,64]. Tre1 and Tre2 have different functions, and Tre1s might have similar functions [15]. In our study, silencing of either *MsTre1* or *MsTre2* led to a significant decrease in the activity of both MsTre1 and MsTre2 (Figure 2D,E). Previous studies have suggested that inhibition of one *Tre* did not reduce the content of trehalose, but might affect the downstream genes’ expression and induce molting defects [23]. In addition, *MsTre1* and *MsTre2* silencing resulted in an increase in trehalose content and a decrease in glucose content in *M. separata* (Figure 2F,G), which is consistent with the study on the interference of *Tre* in *Leptinotarsa decemlineata* [35].

The chitin synthesis pathway is essential for molting of insects [67]. In *N. lugens*, the expression of *CHS1*, *CHS1a*, *CHS1b*, *HK*, *GFAT*, *GNAT*, *PGM*, and *UAP* significantly decreased at 48 h after injection of ds*NlTre* [68]. In our study, the expression of *MsHK* and *MsG6PI* decreased significantly at 6 h (Figure 3C), and the expression of *MsUAP* and *MsCHSA* decreased significantly at 48 h after RNAi of ds*MsTre1* (Figure 3F). The expression of *MsHK*, *MsG6PI*, and *MsCHSB* decreased significantly at 12 h after injection of ds*MsTre2* (Figure 3D). In addition, the expression of *MsUAP*, *MsCHSA*, and *MsCHSB* decreased significantly at 48 h after injection of ds*MsTre2* (Figure 3F). However, the expression of *MsGFAT*, *MsUAP*, and *MsCHSA* was significantly up-regulated at 12 h after injection of ds*MsTre1* and ds*MsTre2* (Figure 3D). These results indicate that the effects of *MsTre1* and *MsTre2* on chitin synthesis-related genes are dynamic; alternatively, there might be a mutual compensatory effect between the functions of *MsTre1* and *MsTre2*. Additional studies are needed to distinguish among the relations among these functions. In addition, our study showed that *MsTre1* silencing resulted in a significant decrease in *MsCht* expression, and *MsTre2* silencing resulted in a significant increase in *MsCht* expression (Figure 3C–F). This is the first study to show that *Tre* has a regulatory effect on *Cht*, and it might be related to changes in the chitin content; however, more research is needed to clarify this possibility.

Chitin is the main component of the peritrophic membrane, trachea, and cuticle in insects [28]. Previous studies have found that the knockdown of *LdTre1a* in *L. decemlineata* induces the death of pupae and reduces the chitin content and the expression of chitin biosynthesis genes [35]. Similarly, we found that injection of both ds*MsTre1* and ds*MsTre2* resulted in a significant reduction in the chitin content of integument and midgut in *M. separata* (Figure 3A,B). Previous studies have noted that there are pronounced differences in the roles of *Tre1* and *Tre2* in regulating the chitin content in insects. *Tre1* is mainly responsible for regulating the expression of *CHSA*, and mainly affects the chitin in integument. *Tre2* mainly regulates the *CHSB* expression, and mainly affects the chitin in midgut [23,64]. There was no significant difference in the effects of *MsTre1* and *MsTre2* on the chitin content in integument and midgut in our study. The results of H&E staining showed that both ds*MsTre1* and ds*MsTre2* delayed the molting of *M. separata*; however, the difference was that no new cuticle appeared when the old cuticle was separated from the epidermis in *M. separata* injected with ds*MsTre1*. In the *M. separata* injected with ds*MsTre2*, the new cuticle appeared simultaneously when the old cuticle was separated from the epidermis (Figure 5A). The TEM results showed that the cuticle of the larvae injected with ds*MsTre1* was thinner than the cuticle of control larvae, and no significant difference in the thickness of the cuticle of larvae injected with ds*MsTre2* and control larvae (Figure 5B). The results indicated that the effects of *MsTre1* and *MsTre2* on *M. separata* molting were not exactly the same, indicating that *MsTre1* had a greater effect on the formation of the cuticle of *M. separata* than *MsTre2*.

Previous studies have found that interference of *Tre* in insects reduces survival rates and results in molting difficulties [33,34]. The silencing of *Tre* in lepidopterans such as *Glyphodes pyloalis*, *Cnaphalocrocis medinalis*, and *S. exigua* results in reduced survival, molting defects, and pupal deformities [69,70,71]. In hemipterans such as *D. citri*, *B. tabaci*, *Sogatella furcifera*, *Laodelphax striatellus*, and *Nilaparvata lugens*, interference of trehalose metabolism regulatory genes reduces the survival rate and results in structural deformities [33,34,72,73]. In our study, the silencing of *MsTre1* and *MsTre2* in *M. separata* led to decreased body weight and length, increased mortality, abnormal phenotypes, and a decreased molting rate (Figure 4), which was consistent with the results of previous research. Silencing of *MsTre1* and *MsTre2* resulted in a significant decrease in the food intake of *M. separata*. Studies of *L. decemlineata* have shown that silencing of *Tre* leads to decreased food intake [35]. Previous studies have suggested that trehalose metabolism affects food selection and consumption by regulating taste receptors and the central nervous system [8]. The decrease in food intake might stem from the decrease in *MsTre1* and *MsTre2* expression and increase in the trehalose content, which increases the difficulty of feeding. More research is needed to clarify the specific feeding behaviors and changes in insect digestion and absorption associated with decreases in *MsTre1* and *MsTre2* expression. In conclusion, MsTre1 and MsTre2 are crucial to the growth and development of *M. separata*, and studies of the function of insect Tre are needed. Additional studies are also needed to optimize RNAi methods and develop Tre inhibitors.

## 5. Conclusions

We cloned and identified *MsTre1* and *MsTre2*. We found that injection of ds*MsTre1* and ds*MsTre2* had significant effects on the larval length, weight, and mortality of *M. separata*, and resulted in abnormal phenotypes. Additionally, the silencing of *MsTre1* and *MsTre2* genes had major effects on the expression of related genes in the trehalose and chitin metabolism pathway, and led to increases in the trehalose and glycogen content, decreases in *MsTre1* and *MsTre2* activity and the glucose content, and decreases in the chitin content. Furthermore, silencing of *MsTre1* severely impaired larval cuticle metabolism; ds*MsTre1*-injected larvae had thinner cuticles with fewer layers than control larvae. These results indicate that *MsTre1* and *MsTre2* play key roles in the growth and survival of *M. separata*; these genes could serve as targets for the control of *M. separata* and aid the development of environmentally friendly pest management strategies.

## Data Availability

Transcriptome Sequencing clean reads in this study were submitted to NCBI SRA database: PRJNA919163.

## Supplementary Materials

The following supporting information can be downloaded at: https://www.mdpi.com/article/10.3390/insects15030142/s1. Figure S1: Sequence alignments between *MsTre1* and *MsTre2*; Figure S2: Phylogenetic tree of insect Tres; Table S1: Details regarding the primers used in this study; Table S2: The cuticle thickness of larvae injected with ds*GFP*, ds*MsTre1*, and ds*MsTre2* was measured under a transmission electron microscope.

## References

[1] Feofilova E.P., Usov A.I., Mysyakina I.S., Kochkina G.A. (2014). Trehalose: Chemical structure, biological functions, and practical application. Microbiology.

[2] Gancedo C., Flores C.L. (2004). The importance of a functional trehalose biosynthetic pathway for the life of yeasts and fungi. FEMS Yeast Res..

[3] Tang B., Wang S., Wang S.G., Wang H.J., Zhang J.Y., Cui S.Y. (2018). Invertebrate *trehalose-6-phosphate synthase* gene: Genetic architecture, biochemistry, physiological function, and potential applications. Front. Physiol..

[4] Friedman S. (1978). Treholose regulation, one aspect of metabolic homeostasis. Annu. Rev. Entomol..

[5] Wyatt G.R. (1967). The biochemistry of sugars and polysaccharides in insects. Adv. Insect Phys..

[6] Elbein A.D., Pan Y.T., Pastuszak I., Carroll D. (2003). New insights on trehalose: A multifunctional molecule. Glycobiology.

[7] Chang Y.P., Zhang B., Du M.F., Geng Z.C., Wei J.Z., Guan R.B., An S.H., Zhao W.L. (2022). The vital hormone 20-hydroxyecdysone controls ATP production by upregulating binding of trehalase 1 with ATP synthase subunit α in *Helicoverpa armigera*. J. Biol. Chem..

[8] Thompson S.N. (2003). Trehalose—The insect ‘blood’ sugar. Adv. Insect Phys..

[9] Becker A., Schlöder P., Steele J.E., Wegener G. (1996). The regulation of trehalose metabolism in insects. Experientia.

[10] Clegg J.S., Evans D.R. (1961). Blood trehalose and flight metabolism in the blowfly. Science.

[11] Candy D.J., Becker A., Wegener G. (1997). Coordination and integration of metabolism in insect flight. Comp. Biochem. Physiol. B Biochem. Mol. Biol..

[12] Avonce N., Mendoza-Vargas A., Morett E., Iturriaga G. (2006). Insights on the evolution of trehalose biosynthesis. BMC Evol. Biol..

[13] Shukla E., Thorat L., Bhavnani V., Bendre A.D., Pal J.K., Nath B.B., Gaikwad S.M. (2016). Molecular cloning and in silico studies of physiologically significant trehalase from *Drosophila melanogaster*. Int. J. Biol. Macromol..

[14] Takiguchi M., Niimi T., Su Z.H., Yaginuma T. (1992). Trehalase from male accessory gland of an insect, *Tenebrio molitor* cDNA sequencing and developmental profile of the gene expression. Biochem. J..

[15] Mitsumasu K., Azuma M., Niimi T., Yamashita O., Yaginuma T. (2005). Membrane-penetrating trehalase from silkworm *Bombyx mori* Molecular cloning and localization in larval midgut. Insect Mol. Biol..

[16] Shukla E., Thorat L.J., Nath B.B., Gaikwad S.M. (2015). Insect trehalase: Physiological significance and potential applications. Glycobiology.

[17] Tang B., Wei P., Chen J., Wang S.G., Zhang W.Q. (2012). Progress in gene features and functions of insect trehalases. Acta. Entomol. Sin..

[18] Bansal R., Mian M.A.R., Mittapalli O., Michel A.P. (2013). Molecular characterization and expression analysis of soluble trehalase gene in *Aphis glycines*, a migratory pest of soybean. Bull. Entomol. Res..

[19] Tan Y., Xiao L.B., Sun Y., Zhao J., Bai L.X., Xiao Y.F. (2014). Molecular characterization of soluble and membrane-bound trehalases in the cotton mirid bug, *Apolygus lucorum*. Arch. Insect Biochem. Physiol..

[20] Tatun N., Singtripop T., Tungjitwitayakul J., Sakurai S. (2008). Regulation of soluble and membrane-bound trehalase activity and expression of the enzyme in the larval midgut of the bamboo borer *Omphisa fuscidentalis*. Insect Biochem. Mol. Biol..

[21] Zhang Q., Lu D.H., Pu J., Wu M., Han Z.J. (2012). Cloning and RNA interference effects of trehalase genes in *Laodelphax striatellus* (Homoptera: Delphacidae). Acta. Entomol. Sin..

[22] Tang B., Yang M.M., Shen Q.D., Xu Y.X., Wang H.J., Wang S.G. (2017). Suppressing the activity of trehalase with validamycin disrupts the trehalose and chitin biosynthesis pathways in the rice brown planthopper, *Nilaparvata lugens*. Pestic. Biochem. Physiol..

[23] Chen J., Tang B., Chen H.X., Yao Q., Huang X.F., Chen J., Zhang D.W., Zhang W.Q. (2010). Different functions of the insect soluble and membrane-bound trehalase genes in chitin biosynthesis revealed by RNA interference. PLoS ONE.

[24] Wang J., He W.B., Su Y.L., Bing X.L., Liu S.S. (2014). Molecular characterization of soluble and membrane-bound trehalases of the whitefly, *Bemisia tabaci*. Arch. Insect Biochem. Physiol..

[25] Zhang J.Z., Zhang X., Arakane Y., Muthukrishnan S., Kramer K.J., Ma E., Zhu K.Y. (2011). Identification and characterization of a novel chitinase-like gene cluster (*AgCht5*) possibly derived from tandem duplications in the African malaria mosquito, *Anopheles gambiae*. Insect Biochem. Mol. Biol..

[26] Zhang W.Q., Chen X.F., Tang B., Tian H.G., Chen J., Yao Q. (2011). Insect chitin biosynthesis and its regulation. Chin. J. Appl. Entomol..

[27] Zhang X., Zhang J.Z., Park Y., Zhu K.Y. (2012). Identification and characterization of two chitin synthase genes in African malaria mosquito, *Anopheles gambiae*. Insect Biochem. Mol. Biol..

[28] Zhu K.Y., Merzendorfer H., Zhang W.Q., Zhang J.Z., Muthukrishnan S. (2016). Biosynthesis, turnover, and functions of chitin in insects. Annu. Rev. Entomol..

[29] Cohen E. (2001). Chitin synthesis and inhibition: A revisit. Pest Manag. Sci..

[30] Nakabachi A., Shigenobu S., Miyagishima S. (2010). Chitinase-like proteins encoded in the genome of the pea aphid, *Acyrthosiphon pisum*. Insect Mol. Biol..

[31] Quan G.X., Ladd T., Duan J., Wen F.Y., Doucet D., Cusson M., Krell P.J. (2013). Characterization of a spruce budworm chitin deacetylase gene: Stage- and tissue-specific expression, and inhibition using RNA interference. Insect Biochem. Mol. Biol..

[32] Xi Y., Pan P.L., Ye Y.X., Yu B., Zhang C.X. (2014). Chitin deacetylase family genes in the brown planthopper, *Nilaparvata lugens* (Hemiptera: Delphacidae). Insect Mol. Biol..

[33] Yu H.Z., Huang Y.L., Lu Z.J., Zhang Q., Su H.N., Du Y.M., Yi L., Zhong B.L., Chen C.X. (2021). Inhibition of trehalase affects the trehalose and chitin metabolism pathways in *Diaphorina citri* (Hemiptera: Psyllidae). Insect Sci..

[34] Gong C., Yang Z.Z., Hu Y., Wu Q.J., Wang S.L., Guo Z.J., Zhang Y.J. (2021). Silencing of the *BtTPS* genes by transgenic plant-mediated RNAi to control *Bemisia tabaci* MED. Pest Manag. Sci..

[35] Shi J.F., Xu Q.Y., Sun Q.K., Meng Q.W., Mu L.L., Guo W.C., Li G.Q. (2016). Physiological roles of trehalose in *Leptinotarsa* larvae revealed by RNA interference of trehalose-6-phosphate synthase and trehalase genes. Insect Biochem. Mol. Biol..

[36] Chen L., Pan Q.J., Waqas M.S., Liu T.X. (2020). Morphological traits for sex identification of the oriental armyworm, *Mythimna separata* (Lepidoptera: Noctuidae). J. Integr. Agric..

[37] Chen G.Q., Yang J.M., Sun D., Han X.X., Tian Y., Liu S.M., Jiang J., Che Z.P. (2020). Syntheses and insecticidal activities of some paeonol-based phenylsulfonylhydrazone derivatives against *Mythimna separata* in vivo. Chemistry.

[38] Wang J.D., Chen L.F., Lin D.J., Zhang J.S., Zhao J.H., Xiao D., Wang R., Wang R., Gao S.J. (2019). Molecular cloning, characterization and functional analysis of *GluCl* from the oriental armyworm, *Mythimna separata* Walker. Pestic. Biochem. Physiol..

[39] Wang D.M., Dai A.M., Liu X.L., Li C.H. (2019). Assessment on control effect of veratridine on cotton aphid. Xinjiang Agric. Sci..

[40] Lin D.J., Zhang Y.X., Fang Y., Gao S.J., Wang R., Wang J.D. (2023). The effect of chlorogenic acid, a potential botanical insecticide, on gene transcription and protein expression of carboxylesterases in the armyworm (*Mythimna separata*). Pestic. Biochem. Physiol..

[41] Azhar M., Freed S., Sabir H., Rafique S., Naeem A., Ahmed R. (2023). Effect of sub-lethal and lethal concentrations of the entomopathogenic fungus *Metarhizium anisopliae* Sorokin on detoxification enzymes and demographic parameters of *Mythimna separata* (Walker). Crop Prot..

[42] Chen X.D., Neupane S., Gill T.A., Gossett H., Pelz-Stelinski K.S., Stelinski L.L. (2021). Comparative transcriptome analysis of thiamethoxam susceptible and resistant Asian citrus psyllid, *Diaphorina citri* (Hemiptera: Liviidae), using RNA-sequencing. Insect Sci..

[43] He C., Liang J.J., Yang J., Xue H., Huang M.J., Fu B.L., Wei X.G., Liu S.N., Du T.H., Ji Y. (2023). Over-expression of *CP9* and *CP83* increases whitefly cell cuticle thickness leading to imidacloprid resistance. Int. J. Biol. Macromol..

[44] Yang H.J., Cui M.Y., Zhao X.H., Zhang C.Y., Hu Y.S., Fan D. (2023). Trehalose-6-phosphate synthase regulates chitin synthesis in *Mythimna separata*. Front. Physiol..

[45] Kumar S., Stecher G., Tamura K. (2016). MEGA7: Molecular Evolutionary Genetics Analysis version 7.0 for bigger datasets. Mol. Biol. Evol..

[46] Vandesompele J., De Preter K., Pattyn F., Poppe B., Van Roy N., De Paepe A., Speleman F. (2002). Accurate normalization of real-time quantitative RT-PCR data by geometric averaging of multiple internal control genes. Genome Biol..

[47] Liu X.J., Li J., Sun Y.W., Liang X.Y., Zhang R., Zhao X.M., Zhang M., Zhang J.Z. (2022). A nuclear receptor HR4 is essential for the formation of epidermal cuticle in the migratory locust, *Locusta migratoria*. Insect Biochem Mol. Biol..

[48] He W., Xu W., Fu K., Guo W., Zhang J. (2020). Low Mismatch Rate between Double-Stranded RNA and Target mRNA Does Not Affect RNA Interference Efficiency in Colorado Potato Beetle. Insects.

[49] Tatun N., Singtripop T., Sakurai S. (2008). Dual control of midgut trehalase activity by 20-hydroxyecdysone and an inhibitory factor in the bamboo borer *Omphisa fuscidentalis* Hampson. J. Insect Physiol..

[50] Bradford M.M. (1976). A rapid and sensitive method for the quantitation of microgram quantities of protein utilizing the principle of protein-dye binding. Anal. Biochem..

[51] Zhao L., Yang M.M., Shen Q.D., Liu X.J., Shi Z.K., Wang S.G., Tang B. (2016). Functional characterization of three trehalase genes regulating the chitin metabolism pathway in rice brown planthopper using RNA interference. Sci. Rep..

[52] Steele J.E. (1961). Occurrence of a hyperglycæmic factor in the corpus cardiacum of an insect. Nature.

[53] Ge L.Q., Zhao K.F., Huang L.J., Wu J.C. (2011). The effects of triazophos on the trehalose content, trehalase activity and their gene expression in the brown planthopper *Nilaparvata lugens* (Stål) (Hemiptera: Delphacidae). Pestic. Biochem. Physiol..

[54] Arakane Y., Muthukrishnan S., Kramer K.J., Specht C.A., Tomoyasu Y., Lorenzen M.D., Kanost M., Beeman R.W. (2005). The *Tribolium chitin synthase* genes *TcCHS1* and *TcCHS2* are specialized for synthesis of epidermal cuticle and midgut peritrophic matrix. Insect Mol. Biol..

[55] Reissig J.L., Strominger J.L., Leloir L.F. (1955). A modified colorimetric method for the estimation of *N*-acetylamino sugars. J. Biol. Chem..

[56] Liu W.M., Xie Y.P., Xue J.L., Gao Y., Zhang Y.F., Zhang X.M., Tan J.S. (2009). Histopathological changes of *Ceroplastes japonicus* infected by *Lecanicillium lecanii*. J. Invertebr. Pathol..

[57] Benoit J.B., Lopez-Martinez G., Michaud M.R., Elnitsky M.A., Lee R.E., Denlinger D.L. (2007). Mechanisms to reduce dehydration stress in larvae of the Antarctic midge, *Belgica antarctica*. J. Insect Physiol..

[58] Zhao K.F., Shi Z.P., Wu J.C. (2011). Insecticide-induced enhancement of flight capacity of the brown planthopper *Nilaparvata lugens* Stål (Hemiptera: Delphacidae). Crop Prot..

[59] Zou Q., Wei P., Xu Q., Zheng H.Z., Tang B., Wang S.G. (2013). cDNA cloning and characterization of two trehalases from *Spodoptera litura* (Lepidoptera; Noctuidade). Genet Mol. Res..

[60] Tang B., Chen X.F., Liu Y., Tian H.G., Liu J., Hu J., Xu W.H., Zhang W.Q. (2008). Characterization and expression patterns of a membrane-bound trehalase from *Spodoptera exigua*. BMC Mol. Biol..

[61] Ma L., Dai W., Li X.C., Zhang Y.L., Zhang C.N. (2015). Molecular cloning and expression analysis of soluble and membrane-bound trehalase genes in the cotton bollworm, *Helicoverpa armigera*. J. Asia Pac. Entomol..

[62] Bartel D.P. (2004). MicroRNAs: Genomics, biogenesis, mechanism, and function. Cell.

[63] Guan R.B., Li H.C., Miao X.X. (2017). RNAi pest control and enhanced BT insecticidal efficiency achieved by dsRNA of chymotrypsin-like genes in *Ostrinia furnacalis*. J. Pest Sci..

[64] Tang B., Wei P., Zhao L., Shi Z.K., Shen Q.D., Yang M.M., Xie G.Q., Wang S.G. (2016). Knockdown of five trehalase genes using RNA interference regulates the gene expression of the chitin biosynthesis pathway in *Tribolium castaneum*. BMC Biotechnol..

[65] Zhang X., Wang Y., Zhang S., Kong X., Liu F., Zhang Z. (2021). RNAi-Mediated Silencing of the Chitinase 5 Gene for Fall Webworm (*Hyphantria cunea*) Can Inhibit Larval Molting Depending on the Timing of dsRNA Injection. Insects.

[66] Ghosh S., Kakumani P.K., Kumar A., Malhotra P., Mukherjee S.K., Bhatnagar R.K. (2014). Genome wide screening of RNAi factors of Sf21 cells reveal several novel pathway associated proteins. BMC Genom..

[67] Arakane Y., Specht C.A., Kramer K.J., Muthukrishnan S., Beeman R.W. (2008). Chitin synthases are required for survival, fecundity and egg hatch in the red flour beetle, *Tribolium castaneum*. Insect Biochem Mol. Biol..

[68] Zhang Y.X., Ge L.Q., Jiang Y.P., Lu X.L., Li X., Stanley D., Song Q.S., Wu J.C. (2015). RNAi knockdown of acetyl-CoA carboxylase gene eliminates jinggangmycin-enhanced reproduction and population growth in the brown planthopper, *Nilaparvata lugens*. Sci. Rep..

[69] Tang B., Chen J., Yao Q., Pan Z.Q., Xu W.H., Wang S.G., Zhang W.Q. (2010). Characterization of a trehalose-6-phosphate synthase gene from *Spodoptera exigua* and its function identification through RNA interference. J. Insect Physiol..

[70] Cha W.H., Lee D.W. (2018). RNA interference of trehalose phosphate synthase inhibits metamorphosis and decreases cold tolerance in the diamondback moth, *Plutella xylostella* (L.). J. Asia Pac. Entomol..

[71] Shao Z.M., Ding J.H., Jiang D.L., Liu Z.X., Li Y.J.C., Wang J., Wang J., Sheng S., Wu F.A. (2021). Characterization and functional analysis of *trehalase* related to chitin metabolism in *Glyphodes pyloalis* Walker (Lepidoptera: Pyralidae). Insects.

[72] Xiong K.C., Wang J., Li J.H., Deng Y.Q., Pu P., Fan H., Liu Y.H. (2016). RNA interference of a trehalose-6-phosphate synthase gene reveals its roles during larval-pupal metamorphosis in *Bactrocera minax* (Diptera: Tephritidae). J. Insect Physiol..

[73] Yang M.M., Zhao L.N., Shen Q.D., Xie G.Q., Wang S.G., Tang B. (2017). Knockdown of two trehalose-6-phosphate synthases severely affects chitin metabolism gene expression in the brown planthopper *Nilaparvata lugens*. Pest Manag. Sci..

