# Supplementary material for: Knockdown of the Expression of Two Trehalase Genes with RNAi Disrupts the Trehalose and Chitin Metabolism Pathways in the Oriental Armyworm, Mythimna separata
Source: Insects. 2024 Feb 21;15(3):142. doi: 10.3390/insects15030142 (PMC10971163; doi:10.3390/insects15030142)
Supplement: Supplementary file 1 [file insects-15-00142-s001.zip › Table S1.pdf]

Table S1. Details regarding the primers used in this study

Gene	GenBank accession number	Primer Name	Primer sequence (5'-3')	Tm (° C)	Amplicon size (bp)	Usage
Trehalase1 (*MsTre1*)	MN894706	*MsTre1*-F	CTCCGACGAAATGACAAA	56.8	2303	cDNA cloning
		MsTre1-R	CCCTCAAAAGCGTAGAAA	57.3		
Trehalase2 (*MsTre2*)	MN894707	*MsTre2*-F	TAGTGTAGTGTTGTGCCATTG	56.8	1987	
		MsTre2-R	GTCGGATAACTATTTCACTCAC	56.6		
Trehalase1 (*MsTre1*)	MN894706	*MsTre1*-q-F	TCTACTACTGGGACTCTTTCTG	55.9	127	RT-qPCR
		MsTre1-q-R	CTTCCGTTAGGGATGTGA	55.6		
Trehalase2 (*MsTre2*)	MN894707	*MsTre2*-q-F	ATGTTGCCAGACACTTTGA	56.1	111	
		MsTre2-q-R	CGTTGAGAATGAACCACC	55.6		
Trehalose-6-phosphate Synthase (*MsTPS*)	MN832898	*MsTPS*-q-F	GAACAGGCGAGGAAGCT	55.0	144	
		MsTPS-q-R	ACTCCAGTCCAATCCGAACG	56.2		
Beta-actin (*Msβ-actin*)	GQ856238	*Msβ-actin*-q-F	CGTCCCAAACCAAACAC	56.2	178	
		Msβ-actin-q-R	TCTGACCCATACCAACCA	56.3		
Glyceraldehyde-3-phosphate dehydrogenase (*MsGAPDH*)	HM055756	*MsGAPDH*-q-F	GCTACAGTCGTTGCCATCAA	56.0	150	
		MsGAPDH-q-R	GAGGACGGAGATTTTGTTGC	55.0		
Chitin synthase A (*MsCHSA*)	KT948989	*MsCHSA*-q-F	GGTGATACAGTTCTCCGCTATG	56.1	121	
		MsCHSA-q-R	CTAGCAGAGCGTCTTGAGATAAG	55.8		
Chitin synthase B (*MsCHSB*)	KY348776	*MsCHSB*-q-F	TCCAGGAGGAAGACCATACA	55.0	92	
		MsCHSB-q-R	GTCAGCCTCCTCTTGAAGTTAG	55.0		
Hexokinase (*MsHK*)	MT995930	*MsHK*-q-F	TCCTTGCGTTGGATTTAG	56.5	123	
		MsHK-q-R	CCCCTGTGCCAGTCATT	55.4		
Glucose-6-phosphate isomerase (*MsG6PI*)	OR933577	*MsG6PI*-q-F	CGGACAGTGGAAGGGAT	56.5	120	
		MsG6PI-q-R	AGCTGGTTGGCATAAGG	55.7		
Glutamine--fructose-6-phosphate aminotransferase (*MsGFAT*)	OR933576	*MsGFAT*-q-F	TTGTTCCGTCTGTCCCA	56.9	186	
		MsGFAT-q-R	CAGACAAACGATGAATGCTA	56.8		
Glucosamine-6-phosphate N-acetyltransferase (*MsGNAT*)	OR933578	*MsGNAT*-q-F	TGTCGGCAATGTTACGC	56.4	165	
		MsGNAT-q-R	CCACGCACAGAGCAGTTAT	57.0		
Phosphoacetylglucosamine	OR933574	*MsPGM*-q-F	ACTCAAGGTCACGGTCAA	55.3	182	
		MsPGM-q-R	TCTCAGCCTCAGCATACAC	55.8		

mutase (*MsPGM*)						
UDP-N-acetylglucosamine pyrophosphorylase (*MsUAP*)	OR933575	*MsUAP*-q-F	AAGCGTGGCGTAGAACA	55.2	148	
		MsUAP-q-R	CGACTGCTTCACTTGGGTA	56.0		
Chitinase (*MsCht*)	MG551526	*MsCht*-q-F	GTATTGTGATTTTCGTGGGA	55.2	171	
		MsCht-q-R	GGATGTGACTGAGGTGGAAC	56.2		
Trehalase1 (*MsTre1*)	MN894706	ds*MsTre1*-F	TAATACGACTCACTATAGGGGTGTCCTTTTGTAAGATGCG	57.3	244	RNA interference
		ds*MsTre1*-R	TAATACGACTCACTATAGGGACGGGTTTTGATTGGTG	56.2		
Trehalase2 (*MsTre2*)	MN894707	ds*MsTre2*-F	TAATACGACTCACTATAGGGTCCGACCAAGGCTGACA	57.2	264	
		ds*MsTre2*-R	TAATACGACTCACTATAGGGCGACCACCAGGCACAAT	56.8		
Green fluorescent protein (*GFP*)	MK558050	ds*GFP*-F	TAATACGACTCACTATAGGGAGTGCTTCAGCCGCTAC	57.0	284	
		ds*GFP*-R	TAATACGACTCACTATAGGGCACCTTGATGCCGTTCT	57.8		
