# Supplementary material for: Knockdown of the Expression of Two Trehalase Genes with RNAi Disrupts the Trehalose and Chitin Metabolism Pathways in the Oriental Armyworm, Mythimna separata
Source: Insects. 2024 Feb 21;15(3):142. doi: 10.3390/insects15030142 (PMC10971163; doi:10.3390/insects15030142)
Supplement: Supplementary file 1 [file insects-15-00142-s001.zip › Table S2.pdf]

Table S2. The cuticles thickness of larvae injected with ds*GFP*, ds*MsTre1* and ds*MsTre2* was measured under transmission electron microscope

dsRNA	Cuticle thickness (μm)	*P*-value
ds*GFP*	14.508±0.3808	-
ds*MsTre1*	9.976±0.1743	<0.0001
ds*MsTre2*	13.622±0.2551	0.08928

Data are presented as the mean ± SE.
