# Supplementary figures and images for: Knockdown of the Expression of Two Trehalase Genes with RNAi Disrupts the Trehalose and Chitin Metabolism Pathways in the Oriental Armyworm, Mythimna separata
Source: Insects. 2024 Feb 21;15(3):142. doi: 10.3390/insects15030142 (PMC10971163; doi:10.3390/insects15030142)

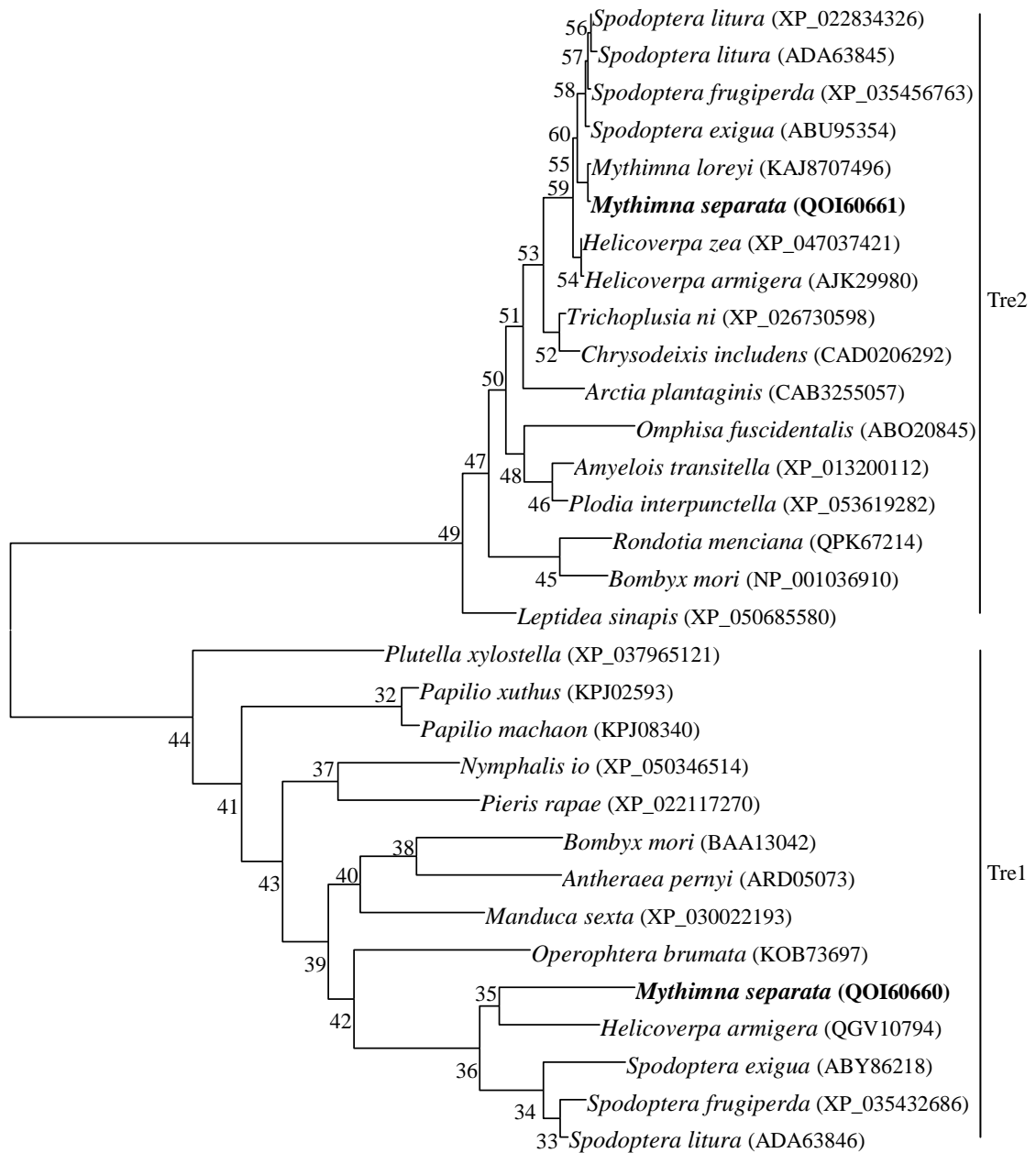

Supplement: Supplementary file 1 [file insects-15-00142-s001.zip › Figure S2.pdf]
